# Granulocytic anaplasmosis in cats from central Europe and molecular characterization of feline *Anaplasma phagocytophilum* strains by *ankA* gene*, groEL* gene and multilocus sequence typing

**DOI:** 10.1186/s13071-023-05954-3

**Published:** 2023-10-06

**Authors:** Anna-Sophia Kruppenbacher, Elisabeth Müller, Matthew L. Aardema, Ingo Schäfer, Friederike D. von Loewenich

**Affiliations:** 1 Institute of Virology, University of Mainz, Mainz, Germany; 2 LABOKLIN GmbH and Co. KG, Bad Kissingen, Germany; 3 Department of Biology, Montclair State University, Montclair, NJ USA; 4 Institute for Comparative Genomics, American Museum of Natural History, New York, NY USA

**Keywords:** *Anaplasma phagocytophilum*, *ankA*, Cat, Europe, Germany, Granulocytic anaplasmosis, *groEL*, Multilocus sequence typing, Switzerland

## Abstract

**Background:**

*Anaplasma phagocytophilum* is a Gram-negative obligate intracellular bacterium that replicates in neutrophil granulocytes. It is transmitted by ticks of the *Ixodes ricinus* complex and causes febrile illness called granulocytic anaplasmosis primarily in humans, horses, dogs, sheep, cattle and goats. In comparison, clinically apparent disease has been described rarely in cats especially compared to dogs and horses. It is currently unknown whether cats are less susceptible to *A. phagocytophilum* or whether granulocytic anaplasmosis might be underdiagnosed in cats.

**Methods:**

To address this question, we examined clinical signs and laboratory findings in seven *A. phagocytophilum* infected cats from Germany and Switzerland. We then genetically characterized feline *A. phagocytophilum* strains and compared them to those from other hosts showing clinically apparent disease. For this purpose, *ankA*-based, *groEL*-based and multilocus sequence typing (MLST) were applied. Furthermore, the concordance between these typing methods was assessed.

**Results:**

Fever, lethargy and anorexia were the most common clinical signs in cats suffering from granulocytic anaplasmosis. The most frequent laboratory finding was thrombocytopenia. All three typing methods consistently indicated that the *A. phagocytophilum* strains found infecting cats are the same as those that cause disease in humans, dogs and horses. In general, the three typing methods applied exhibited high concordance.

**Conclusions:**

The genetic characterization of the feline *A. phagocytophilum* strains indicates that strain divergence is not the explanation for the fact that granulocytic anaplasmosis is much less frequently diagnosed in cats than in dogs and horses. Otherwise, it may be possible that cats are less susceptible to the same strains than dogs and horse are. However, due to the unspecific clinical signs, it should be considered that granulocytic anaplasmosis may be under-diagnosed in cats.

**Graphical Abstract:**

**Figure:**
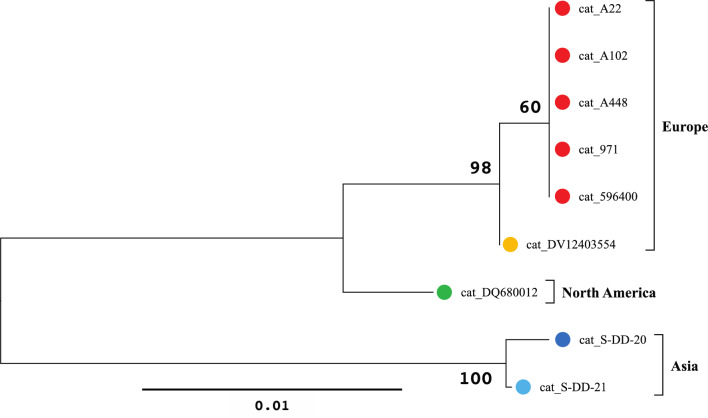

## Background

*Anaplasma phagocytophilum* is a Gram-negative obligate intracellular bacterium that replicates in neutrophil granulocytes [1]. It is transmitted to humans and animals by ticks of the *Ixodes ricinus* complex [2]. The main vector in Europe is *I. ricinus* [3]. *Anaplasma phagocytophilum* causes febrile illness primarily in humans [2], domestic animals such as horses [4], dogs [5] and cats [6], and farm animals such as sheep, cattle and goats [7]. The disease is called granulocytic anaplasmosis in humans and domestic animals and tick-borne fever in ruminants.

*Anaplasma phagocytophilum* is widely distributed globally, having been detected in the Americas, Europe, Asia and Africa [8]. However in cats, clinically apparent disease together with a consistent molecular identification of the infecting agent has been rarely described. Well-documented feline cases have been reported from Austria [9], Finland [10], Germany [9, 11, 12], Poland [13, 14], Sweden [15], Switzerland [9], the UK [16] and the USA [17, 18].

The clinical signs of feline granulocytic anaplasmosis are unspecific and most often comprise fever, lethargy and anorexia [19]. Typical laboratory findings are thrombocytopenia, lymphopenia and anemia [19]. The diagnosis is made best by amplification of pathogen-specific DNA from EDTA-anticoagulated blood [20] followed by sequencing of the amplicon to ensure specificity [6]. Usage of the *msp2* gene as a molecular target is discouraged because a significant proportion of *msp2* PCR results are false positives for currently unknown reasons [21]. The microscopic detection of bacterial inclusions in granulocytes, so-called morulae, in Giemsa-stained blood smears is possible but less sensitive and observer-dependent [22]. Although widely performed, serology is useless in patients presenting with an acute *A. phagocytophilum* infection because most are seronegative at this time point [20, 23].

The genetic characterization of *A. phagocytophilum* strains by various single and multilocus sequence typing schemes has revealed an association of specific types with the host species, vector species and geographic origin [24, 25]. However to date, it is not possible to clearly determine which of these three interdependent variables has the biggest impact due to huge sampling biases, the variety of typing methods applied and sequence length variation.

Presently, information on the *A. phagocytophilum* types infecting cats is limited. Strains from four European cats suffering from granulocytic anaplasmosis were characterized using multilocus sequence typing (MLST) and *ankA*-based typing [26, 27]. The *A. phagocytophilum* strains detected in three asymptomatic cats from Italy were subjected to *groEL*-based typing [28]. Furthermore, genetic characterization of strains identified in two asymptomatic cats from Korea relied on the sequencing of *groEL* and *msp2* gene fragments [29]. Additionally, one *groEL* sequence from a feline host originating from the USA was part of the study by Jaarsma et al., but information on the disease status of the animal was not provided [30].

Thus, *A. phagocytophilum* strains eliciting clinical disease in cats have been characterized only rarely. Furthermore, several target genes and target gene sequences of differing length have been used in the past, which hinders the ability to compare results. Therefore, we attempted to genetically characterize *A. phagocytophilum* strains from seven cats suffering from granulocytic anaplasmosis using typing schemes that have been previously applied in cats (*ankA* and *groEL* gene-based typing, MLST) and that are highly standardized and curated (MLST).

## Methods

### Clinical signs of feline granulocytic anaplasmosis

The clinical data and laboratory findings of seven cats suffering from granulocytic anaplasmosis were part of a previously published study [9] but are reported here in more detail. Breed, sex, age, country of origin, clinical signs, rectal temperature, complete blood cell counts and year of detection were recorded. In some cats, electrolytes (sodium, potassium, phosphorus), transaminases (alanine aminotransferase, aspartate aminotransferase), alkaline phosphatase, bilirubin, creatinine and urea were also measured. Complete blood cell counts were determined using the ADVIA 2120i (Siemens Healthineers, Erlangen, Germany) or the Sysmex XT-2000i (Sysmex Deutschland, Norderstedt, Germany) instruments. Platelet counts < 90 × 10^9^/l were confirmed by manual counting using a hemocytometer. Giemsa-stained blood smears were investigated microscopically for morulae at a magnification of 10 × 100. Electrolytes, transaminases, alkaline phosphatase, bilirubin, creatinine and urea were determined using the Cobas 8000 (Roche Diagnostics, Mannheim, Germany).

### Genetic characterization

DNA samples positive for *A. phagocytophilum* were isolated from EDTA-blood drawn between the years 2018 and 2020 from seven cats suffering from granulocytic anaplasmosis. 523 bp (without primers) of the *ankA* gene [26] and 530 bp (without primers) of the *groEL* gene [31] were amplified and sequenced bi-directionally as described. For the purpose of this publication, *ankA* allele numbers were assigned to all applicable sequences published previously. The *ankA* cluster was determined as defined by Huhn et al. [26]. g*roEL* haplotypes of the long fragment and *groEL* clusters were assigned according to the nomenclature defined by Jaarsma et al. [30]. The PubMLST database (https://pubmlst.org/) was updated to contain not only the MLST allele definitions but also the *ankA* allele and the *groEL* long-fragment haplotype nomenclature. For MLST, seven housekeeping genes (*pheS, glyA, fumC, mdh, sucA, dnaN* and *atpA*) were amplified and sequenced bi-directionally as reported previously [26]. Different sequences of a given locus were ascribed a unique but arbitrary allele number, and each unique combination of alleles was assigned a sequence type (ST). Clonal complexes (CC) were defined by sharing identical alleles at five of the seven loci with at least one other member of the group. MLST clusters were defined as described previously [26, 27].

Sequences were aligned by ClustalW applying the IUB matrix. Pairwise distances were calculated using MEGA 11.0.13 [32]. The phylogenetic tree was constructed using the neighbor-joining method with the Jukes-Cantor model in the program MEGA 11.0.13. Bootstrap analysis was conducted with 1000 replicates. To test for the concordance between different typing methods, adjusted Wallace coefficients [33] were calculated using the online tool accessible at: http://www.comparingpartitions.info/index.php?link=Tool.

The nucleotide sequences are available at GenBank under the accession numbers OQ435068–OQ435074 (*ankA*), OQ435150–OQ435152 (*groEL*), OQ435080–OQ435084 (*pheS*), OQ435090–OQ435094 (*glyA*), OQ435100–OQ435104 (*fumC*), OQ435110–OQ435114 (*mdh*), OQ435120–OQ435124 (*sucA*), OQ435130—OQ435134 (*dna*N) and OQ435140–OQ435144 (*atpA*).

## Results

### Clinical signs of feline granulocytic anaplasmosis

Breed, sex, age, country of origin, clinical signs and rectal temperature of the seven cats are shown in Table 1. The median age of the cats was 10.0 years. Six cats lived in Germany and one in Switzerland. Information on clinical signs and rectal temperature was available in six out of seven cats. The most common clinical signs were lethargy and anorexia. All six cats had an increased body temperature.

**Table 1:** Breed, sex, age, country of origin, clinical signs and rectal temperature of the seven cats

Animal	Breed	Sex	Age (years)	Country	Clinical signs	Rectal temperature (°C) ^a^
cat_A4	LaPerm Longhair	Male^b^	7	Germany	Lethargy, anorexia	40.8
cat_A22	European Shorthair	Male^b^	0.8	Germany	Lethargy, vomitus, diarrhea	40.4
cat_A102	Mixed	Unknown	8	Germany	Unknown	Unknown
cat_A448	European Shorthair	Female^c^	14	Germany	Lethargy, anorexia	40.6
cat_A449	European Shorthair	Female^c^	13	Germany	Lethargy, anorexia	39.6
cat_A480	European Shorthair	Female^c^	3	Germany	Lethargy, anorexia	39.2
cat_A512	European Shorthair	Male^b^	12	Switzerland	Lethargy, anorexia	40.1

^a^Norm: 36.7–38.9 °C [34]

^b^Neutered

^c^Spayed

Complete blood cell counts of the seven cats are shown in Table 2. The most common laboratory finding was thrombocytopenia, which was present in five cats. The thrombocytopenia was mild in three and moderate in two animals. Leukopenia was observed in two, lymphopenia in four and anemia in one cat. Three animals showed a mild to moderate leukocytosis. Morulae inside neutrophils were present in the blood smears of five cats.

**Table 2:** Complete blood cell counts of the seven cats

Parameter	Reference values^a^	cat_A4	cat_A22	cat_A102	cat_A448	cat_A449	cat_A480	cat_A512
Hemoglobin (g/l)	90–150	117	106	128	129	92	51	125
Hematocrit (l/l)	0.3–0.44	0.35	0.37	0.41	0.44	0.31	0.19	0.43
Red blood cell count (× 10^12^/l)	5.0–10.0	5.11	7.22	8.66	8.32	6.68	3.07	8.5
Thrombocytes (× 10^9^/l)	180–550	78	171	82	209	191	105	130
White blood cell count (× 10^9^/l)	6.0–11.0	5.11	10.0	4.1	23.4	8.1	11.8	20.6
Lymphocytes (× 10^9^/l)	1.0–4.0	0.1	ND ^b^	0.3	0.5	0.3	3.8	1.0
Monocytes (× 10^9^/l)	0.04–0.5	0.22	ND	0.1	0	0	0	0
Segmented neutrophils (× 10^9^/l)	3.0–11.0	IR ^c^	ND	3.6	22.2	8.7	7.8	18.7
Band neutrophils (× 10^9^/l)	< 0.6	0.05	ND	0	0.7	0	0	0.8
Eosinophils (× 10^9^/l)	0.04–0.6	0.02	ND	0	0	0	0.2	0
Neutrophils with morulae	NA ^d^	+	-	+	+	+	-	+

^a^According to LABOKLIN GmbH and Co. KG, Bad Kissingen, Germany

^b^ND not done

^c^IR inconclusive result

^d^NA not applicable

When tested, electrolytes, transaminases, alkaline phosphatase, bilirubin, creatinine and urea were within normal ranges (data not shown) with the exception of a moderate hyperbilirubinemia of 15.2 µmol/l (normal range < 3.4 µmol/l) in cat A480.

### *ankA* gene

The *ankA* gene could be amplified from the blood of all seven cats. The obtained sequences were compared to four sequences from cats (GenBank accession numbers GU236864, FJ515309, MH987707, MH987708) published previously [26, 27] and to one unpublished sequence from a cat available at GenBank (GenBank accession number OQ435075). All 12 feline sequences originated from Europe and were part of *ankA* cluster 1 [26, 27]. They were 99.2–100% identical to each other and belonged to the *ankA* alleles 12 (9/12), 13 (1/12), 14 (1/12) and 15 (1/12) as shown in Table 3.

**Table 3:** *ankA* cluster, *ankA* allele, *groEL* cluster, *groEL* haplotype, MLST cluster, clonal complex (CC), sequence type (ST) and housekeeping gene allele numbers of the *Anaplasma phagocytophilum* strains from the seven cats that were part of the present study and from eight cats with sequences available at GenBank

Animal	*ankA* cluster	*ankA *allele	*groEL* cluster	*groEL* haplotype	MLST cluster	CC	ST	*pheS*	*glyA*	*fumC*	*mdh*	*sucA*	*dnaN*	*atpA*
cat_A4^a^	1	12	ND^c^	ND	1	1	188	27	15	6	2	2	11	57
cat_A22^a^	1	12	1	6	1	1	25	27	15	6	2	2	11	7
cat_A102^a^	1	12	1	6	1	1	25	27	15	6	2	2	11	7
cat_A448^a^	1	12	1	6	1	1	25	27	15	6	2	2	11	7
cat_A449^a^	1	12	ND	ND	ND	ND	ND	ND	ND	ND	ND	ND	ND	ND
cat_A480^a^	1	12	ND	ND	ND	ND	ND	ND	ND	ND	ND	ND	ND	ND
cat_A512^a^	1	12	ND	ND	1	1	25	27	15	6	2	2	11	7
cat_1^b^	1	12	ND	ND	1	1	25	27	15	6	2	2	11	7
cat_2^b^	1	13	ND	ND	1	1	55	27	15	6	3	2	27	7
cat_971^b^	1	14	1	6	1	NT ^d^	242	27	33	6	55	2	66	61
cat_596400^b^	1	12	1	6	1	1	25	27	15	6	2	2	11	7
cat_DV12403554^b^	1	15	1	5	1	1	55	27	15	6	3	2	27	7
cat_S-DD-20^b^	ND	ND	4	178	ND	ND	ND	ND	ND	ND	ND	ND	ND	ND
cat_S-DD-21^b^	ND	ND	4	179	ND	ND	ND	ND	ND	ND	ND	ND	ND	ND
cat_DQ680012^b^	ND	ND	1	113	ND	ND	ND	ND	ND	ND	ND	ND	ND	ND

^a^This study

^b^Sequences available at GenBank

^c^ND not done

^d^NT nontypeable

### *groEL* gene

The *groEL* gene could be amplified only from three of the seven cats as the extracted DNA from the other four had been previously depleted. The obtained sequences were compared to three unpublished sequences from cats available at GenBank (OQ435153, OQ656703 and OQ656704) and to three sequences from cats (GenBank accession numbers KU519284, KU519285 and DQ680012) published previously [29, 30]. The *groEL* sequence with the GenBank accession number KU712086 (isolate 971) probably originates from the same animal as the sequence with the GenBank accession number OQ656704 (cat_971). However, the information concerning the country of origin (Finland versus Germany) and the disease state (asymptomatic carrier versus granulocytic anaplasmosis) is conflicting [35]. Here, only the sequences from cat_971 were considered for analysis. Unfortunately, three feline sequences reported by Balboni et al. [28] were too short to be included (232 bp–520 bp). Thus, nine *groEL* sequences of feline origin were analyzed.

Six sequences originated from Europe, two were from Asia and one from North America. Seven sequences belonged to the *groEL* cluster 1 [30], six of which were from Europe and one from North America. The two sequences from Asia were part of *groEL* cluster 4 [30]. The nine *groEL* sequences were 95.8–100% identical to each other and belonged to the *groEL* long-fragment haplotypes 5 (1/9), 6 (5/9), 113 (1/9), 178 (1/9) and 179 (1/9) as shown in Table 3.

**Fig. 1:**
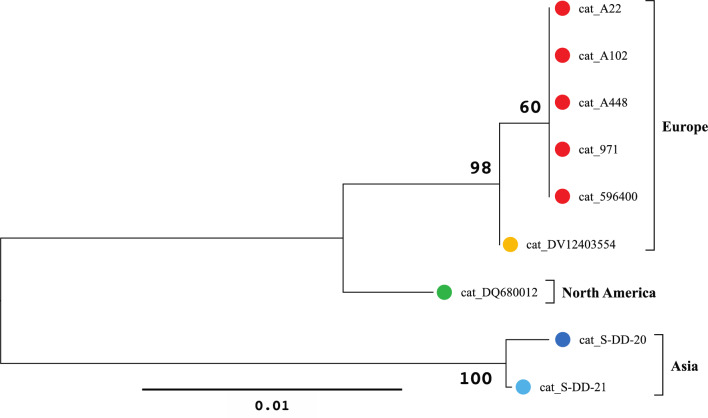
Phylogenetic tree calculated from the nine feline *groEL* sequences. The data set contained 530 positions. Tree construction was achieved by the neighbor-joining method using the Jukes-Cantor matrix. Bootstrap values are shown next to the branches. The scale bar indicates the number of nucleotide substitutions per site. Animals with identical *groEL* haplotype are shown in the same color

The seven sequences from Europe and North America (*groEL* cluster 1) had an identity of 98.9–100% to each other and the two sequences from Asia (*groEL* cluster 4) of 99.8%, respectively. Thus, the feline *groEL* sequences from Europe and North America were more similar to each other than those from Asia, as shown in Fig. 1.

### MLST

All seven housekeeping genes could be amplified only from five of the seven cats as the extracted DNA from the other two had been previously depleted. After sequencing, the sequence type (ST) was ascribed as outlined in the Methods section. The respective STs were compared to four STs from feline *A. phagocytophilum* strains published previously [26, 27] and using unpublished sequences from one cat available at GenBank (OQ435085, OQ435095, OQ435105, OQ435115, OQ435125, OQ435135 and OQ435145). All ten STs of feline origin belonged to the MLST cluster 1 [26, 27]. The ten concatenated housekeeping gene sequences (2877 bp) were 99.5–100% identical to each other. ST 25 was found in six, ST 55 in two and ST 188 and ST 242 in one animal, respectively (Table 3). ST 25, 55 and 188 belong to clonal complex (CC) 1 [26, 27].

### Concordance between typing methods

The concordance between *ankA*, *groEL* and MLST cluster was 100% (Table 3). Adjusted Wallace coefficients [33] were calculated to compare the partitioning of *ankA* alleles, *groEL* haplotypes and ST using the six *A. phagocytophilum* strains with complete information (Table 4). The concordance between *ankA* allele and *groEL* haplotype and *ankA* allele and ST was 100%. The concordance between ST and *ankA* allele and between ST and *groEL* haplotype was 100% as well. The concordance between *groEL* haplotype and *ankA* allele and between *groEL* haplotype and ST was only 33.3% as one of the two haplotypes found (haplotype 6) was present in 83% of the samples.

**Table 4:** Adjusted Wallace coefficients and 95% confidence intervals (in parentheses) in percent indicating the concordance between the partitions *ankA* allele, *groEL* haplotype and ST for the six *Anaplasma phagocytophilum* strains with complete information

Partition	*ankA* allele	*groEL* haplotype	ST
*ankA* allele	–	**100** (100–100)	**100** (100–100)
*groEL* haplotype	33.3 (0.0–100)	–	33.3 (0.0–100)
ST	**100** (100–100)	**100** (100–100)	–

Adjusted Wallace coefficients > 75% are marked in bold

The association between disease state and typing method could not be calculated because there was a strong bias regarding geographic origin as the two asymptomatic carriers were both from Asia (Table 5).

**Table 5:** *groEL* cluster, *groEL* haplotype, disease state, country and continent of origin, year of detection and source of detection of the *Anaplasma phagocytophilum* strains from the seven cats that were part of the present study and from eight cats with sequences available at GenBank

Animal	*groEL* cluster	*groEL* haplotype	Disease state	Country	Continent	Year	Source
cat_A4^a^	ND^d^	ND	Granulocytic anaplasmosis	Germany	Europe	2018	Blood
cat_A22^a^	1	6	Granulocytic anaplasmosis	Germany	Europe	2018	Blood
cat_A102^a^	1	6	Granulocytic anaplasmosis	Germany	Europe	2018	Blood
cat_A448^a^	1	6	Granulocytic anaplasmosis	Germany	Europe	2020	Blood
cat_A449^a^	ND	ND	Granulocytic anaplasmosis	Germany	Europe	2020	Blood
cat_A480^a^	ND	ND	Granulocytic anaplasmosis	Germany	Europe	2020	Blood
cat_A512^a^	ND	ND	Granulocytic anaplasmosis	Switzerland	Europe	2020	Blood
cat_1^b^	ND	ND	Granulocytic anaplasmosis	Austria	Europe	2003	Blood
cat_2^b^	ND	ND	Granulocytic anaplasmosis	Switzerland	Europe	2008	Blood
cat_971^b, c^	1	6	Granulocytic anaplasmosis	Germany	Europe	2008	Blood
cat_596400^b^	1	6	Granulocytic anaplasmosis	Germany	Europe	2014	Blood
cat_DV12403554^b^	1	5	Granulocytic anaplasmosis	Switzerland	Europe	2021	Blood
cat_S-DD-20^b^	4	178	Asymptomatic carrier	South Korea	Asia	2012–2015	Blood
cat_S-DD-21^b^	4	179	Asymptomatic carrier	South Korea	Asia	2012–2015	Blood
cat_DQ680012^b^	1	113	Unknown	USA	North America	2004	Blood

^a^This study

^b^Sequences available at GenBank

^c^Conflicting information concerning disease state and country

^d^ND not done

The concordance between *groEL* cluster, *groEL* haplotype and continent was analyzed for the nine strains with known *groEL* haplotype. The concordance between *groEL* cluster and continent was 50.9% as *groEL* cluster 1 was found in cats from Europe and North America whereas *groEL* cluster 4 was restricted to the two animals from Asia (Tables 5, 6). Vice versa, the concordance between continent and *groEL* cluster was 100% (Table 6).

**Table 6:** Adjusted Wallace coefficients and 95% confidence intervals (in parentheses) in percent indicating the concordance between the partitions *groEL* cluster, *groEL* haplotype and continent of origin for the nine *Anaplasma phagocytophilum* strains with complete information

Partition	*groEL* cluster	*groEL* haplotype	Continent
*groEL* cluster	–	24.5 (0.0–80.0)	50.9 (0.0–100)
*groEL* haplotype	**100** (100–100)	–	**100** (100–100)
Continent	**100** (100–100)	48.1 (0.0–100)	–

Adjusted Wallace coefficients > 75% are marked in bold

The concordance between *groEL* haplotype and continent was 100% because the respective haplotypes found were continent-specific. However, only one sample from North America and two samples from Asia were part of the analysis, whereas all others were from Europe.

## Discussion

In the present study, fever, lethargy and anorexia were the most common clinical signs in cats suffering from granulocytic anaplasmosis. Furthermore, the most frequent laboratory finding was thrombocytopenia. Thus, clinical and laboratory observations were in line with the literature [19].

Detailed medical records were available only in seven cats. For six cats, the disease state could be extracted from GenBank or from the literature. In two cats, this information was conflicting or missing. Only two asymptomatic carriers were part of the study, and both were from Asia. Thus, due to the small number of cases analyzed here and the substantial sampling bias, it was impossible to correlate distinct clinical or laboratory observations or the disease state with certain *A. phagocytophilum* types.

The 12 European feline *ankA* sequences analyzed here belonged to *ankA* cluster 1. *ankA* cluster 1 has been reported previously [26, 27] to contain, among others, *A. phagocytophilum* strains from humans, domestic animals (horses, dogs), farm animals (sheep, cattle, goats), wildlife (European bison, red deer, red foxes, wild boar) and small mammals (European hedgehog). The *ankA* cluster 1 was initially described to harbor sequences from Europe and North America [26, 27] but is now restricted to strains from Europe since North American *ankA* clusters 11 and 12 were separated from cluster 1 [27]. The *ankA* alleles 12, 13, 14 and 15 found here in cats were detected previously in samples from humans, dogs, horses and other hosts [26, 27].

The nine feline *groEL* sequences analyzed here belonged to *groEL* cluster 1. *groEL* cluster 1 has been previously described [30] to contain *A. phagocytophilum* strains from humans, domestic animals (horses, dogs), farm animals (sheep, cattle, goats), wildlife (moose, red deer, roe deer, red foxes, wild boar) and small mammals (European hedgehog, Northern white-breasted hedgehog), among others. Sequences belonging to *groEL* cluster 1 were reported to be restricted to Europe and the Americas [30].

The two sequences from Asia belonged to *groEL* cluster 4. *groEL* cluster 4 has been described previously [30] to harbor *A. phagocytophilum* strains from a human, two dogs, two goats, rodents and ticks [30]. *groEL* cluster 4 is restricted to strains from Asia. However, it was previously reported to harbor strains from Europe as well [30], because strains from the Asian part of Russia were erroneously ascribed to the European continent.

The *groEL* long-fragment haplotypes 5, 6, 113, 178 and 179 found here in cats were detected previously, among others, in samples from humans, dogs and horses [30].

All ten STs of feline origin belonged to the MLST cluster 1. MLST cluster 1 has been previously reported [26, 27] to contain *A. phagocytophilum* strains from different hosts which included humans, domestic animals (horses, dogs), farm animals (sheep, cattle, goats), wildlife (European bison, red deer, red foxes, wild boar) and small mammals (European hedgehog). The MLST cluster 1 harbors strains from Europe and North America [26, 27]. ST 25 and ST 55 found here in cats were previously detected in samples from humans, dogs and horses as well as from other hosts [26, 27].

Thus, *ankA*-based typing, *groEL*-based typing and MLST showed consistent results. Specifically, the *A. phagocytophilum* strains found to infect cats were the same that caused disease in humans, dogs and horses. This was reported before for four cats (cat_1, cat_2, cat_971 and cat_596400) regarding *ankA*-based typing and MLST [26, 27] and for three cats (cat_S-DD-20, cat_S-DD-21, cat_DQ680012) regarding *groEL*-based typing [30] but is confirmed here on a broader data basis including a direct comparison of the three typing methods. Thus, strain divergence of feline strains is unlikely to explain the fact that granulocytic anaplasmosis is much less frequently diagnosed in cats than in dogs and horses. Therefore, due to the unspecific clinical signs, it should be considered that granulocytic anaplasmosis might be under-diagnosed in cats. However, it is also possible that cats are less susceptible to the same strains than dogs and horses. Differences in animal behavior between dogs and cats such as efficient removal of ticks by self-grooming cats might also serve as an explanation [36].

The observation that the same *A. phagocytophilum* strains infect cats, humans, dogs and horses was true for Europe, North America and Asia. However, feline strains from Europe and North America were more similar to each other than to those from Asia according to the *groEL*-based typing, although the overall identity was high with 95.8–100%. This result has to be confirmed by further studies with higher sample numbers as there was a substantial sampling bias regarding the continent of origin. Considering only *A. phagocytophilum* strains from non-vector species, the *groEL* data base contains 669 strains from Europe, 25 strains from the Americas and 25 strains from Asia [30]. The same is true for the *ankA*-based typing with 469 strains from Europe, 25 strains from North America and 7 strains from Asia and for MLST with 386 strains from Europe, 18 strains from North America and 7 strains from Asia [26, 27].

Unfortunately, three feline *groEL* sequences reported by Balboni et al. [28] were too short to be included. This underlines the importance of standardizing the typing of *A. phagocytophilum* strains to ensure the comparability of results across studies. Furthermore, during our analysis, it was noticed that samples from the Asian part of Russia were erroneously ascribed to the European continent by Jaarsma et al. [30] and that conflicting information concerning country of origin and disease state was reported for cat_971 [26, 35]. Therefore, we further emphasize the importance of collecting and reporting epidemiological data as completely and correctly as possible. Aliases of sample names should be avoided or correctly documented to prevent the same strain being considered multiple times within an analysis.

In our opinion, the typing of *A. phagocytophilum* strains should concentrate on target genes that already have substantial information available concerning sample numbers, different host species and diverse geographic origin. The same fragment length and position must also be used. Curated databases available via the internet should ensure quality control, correctness and completeness of the data and a universal nomenclature. At least, host species, country of origin, year, site of detection and disease state should be reported. To facilitate the accessibility of the data, the PubMLST database (https://pubmlst.org/) was updated to contain not only the MLST allele definitions, but also the *ankA* allele and *groEL* long-fragment haplotype nomenclature. Scientists are invited to submit their typing and isolates data to this database.

## Conclusions

Although our analysis was limited by small sample numbers, we nonetheless provide important information on the clinical signs of cats suffering from granulocytic anaplasmosis. The genetic characterization using *ankA*-based typing, *groEL*-based typing and MLST showed that cats are infected by the same *A. phagocytophilum* strains as humans, dogs and horses. Given the sparse reports of granulocytic anaplasmosis in cats, feline infection by *A. phagocytophilum* might be under-diagnosed. However, the possibility remains that cats might be less susceptible to the same strains than dogs and horses are. Increased disease awareness in feline hosts should help to answer this question in the future.

## Data Availability

All data generated or analyzed during this study are included in this published article. All nucleotide sequences are available at GenBank. *ankA* allele, *groEL* haplotype, MLST profile and epidemiological information of the samples was submitted to the *Anaplasma phagocytophilum* isolates database hosted on PubMLST (https://pubmlst.org/).

## Abbreviations

CC: Clonal complex
MLST: Multilocus sequence typing
ST: Sequence type
